# In the COVID-19 era, let’s keep an eye on clinical trials in Africa

**DOI:** 10.7189/jogh.10.020312

**Published:** 2020-12

**Authors:** Rafiou Agoro

**Affiliations:** Department of Medical and Molecular Genetics, Indiana University School of Medicine, Indianapolis, Indiana, USA

Research on human has been a critical pillar for the advancement of medical knowledge including the development of novel vaccines and drugs. Yet the legacy of scientific experiments on human is far to be without controversies. Unethical scientific experiments have been conducted on enslaved persons, World War II prisoners, and even in postwar period where cases of unethical experiments were reported on prisoners, mentally disabled, and ethnic minorities such us the Tuskegee Syphilis study [1] conducted between 1932 and 1972 in the United States. In Africa, suspected unethical clinical trials have been reported. For example, in 2001, the pharmaceutical company Pfizer has been accused over informed consent issues and unethical trial in Nigeria during the meningitis epidemic in 1996 [2].

To prevent unethical practices in medical research, the World Medical Association (WMA) through the Declaration of Helsinki has established since 1964 guidelines for medical researchers to balance the need to generate medical knowledge with the need to protect the health and interests of research participants [3]. In 1978, following the infamous Tuskegee Syphilis Study, the National Commission for the Protection of Human Subjects of Biomedical and Behavioral Research of the United States has stated moral principles and guidelines for the protection of human subjects summarized in The Belmont Report and available as DHEW (Department of Health Education and Welfare) Publication No. (OS) 78-0013 and No. (OS) 78-0014 [4,5].

## COVID-19 AND VACCINE AND MEDICINE DEVELOPMENT

Still counting, it has been several months since the COVID-19 (novel coronavirus, also known as 2019-nCoV or SARS-CoV-2) outbreak disrupts the world [6]. One of the evident solutions against this outbreak is the development of an effective vaccine and/or a medicine to protect our human community. While waiting for an effective treatment, worldwide we are all experiencing uncomfortable rigor policies such as cities and countries lockdowns coupled with social distancing policies and the use of personal protective equipment. All these new normal realities are not for sure our main expectations when we kicked off a new decade with lot of promises.

Due to the high contagiousness of COVID-19 [7] and the lack of an effective medicine, governments and companies are urging their teams to develop new vaccine and therapy with the noble mission to protect us from COVID-19 and preserve our economic achievements. In view of the current global pandemic situation, as a young scientist, I can understand the pressure my colleagues from public and private institutions are facing to develop rapidly vaccine and medicine. It is definitively a tough race.

The traditional workflow to develop drugs and vaccines takes from several months to years before reaching the market. Throughout preclinical experiments, scientists determine the safety and effectiveness of candidate vaccines and drugs. After this first step, several phases of clinical trials are followed by enrolling patients and volunteers to participate in small pilot studies to confirm the safety of the treatment protocol before scaling up to large cohort studies for further confirmatory tests. During clinical trials, one of the responsibilities of scientists is to determine the appropriateness of clinical trial parameters such as randomization in addition to the design of a risk-free placebo control especially during disease outbreaks with high contagiousness and mortality rate. Other responsibilities from scientists are to ensure that the proposed methodologies of the trial are consented by the participative communities.

There is no doubt that scientists are trained with strong ethical values. However, under the current pandemic circumstances, we cannot exclude the possibilities that some scientists violate clinical trials rules and guidelines to accelerate new vaccine and medicine development. For instance, identifying countries with weak clinical trial guidelines and policies could be preferred for unethical and fast clinical trials. Currently, there is no uniform policy for conducting trials across the world, this means every government is responsible to establish their own policies. Low-income countries, notably Africans, could be preferred as a basis for experimentations on human subjects due to the lack of stringent policies in comparison to their high-income counterparts.

During clinical trials, healthy volunteers, and sick participants who are often powerless, expect from scientists and physicians to incorporate strong ethics and bioethics rules in their experimental design. From their governments, these participants expect an establishment of critical and stringent guidelines and recommendations for an ethically acceptable trial. It is the responsibility of the governments to fully understand the risks of clinical trials on their population in the goal to prevent unwanted and unwarranted setbacks especially during the current pandemic situation.

Under the current pandemic circumstances, our moral rationale could be seriously challenged as scientists; however, these moments could represent an opportunistic timeframe for some governments to definitely upgrade their clinical trial policies and guidelines because of ongoing and upcoming clinical trials.

**Figure:**
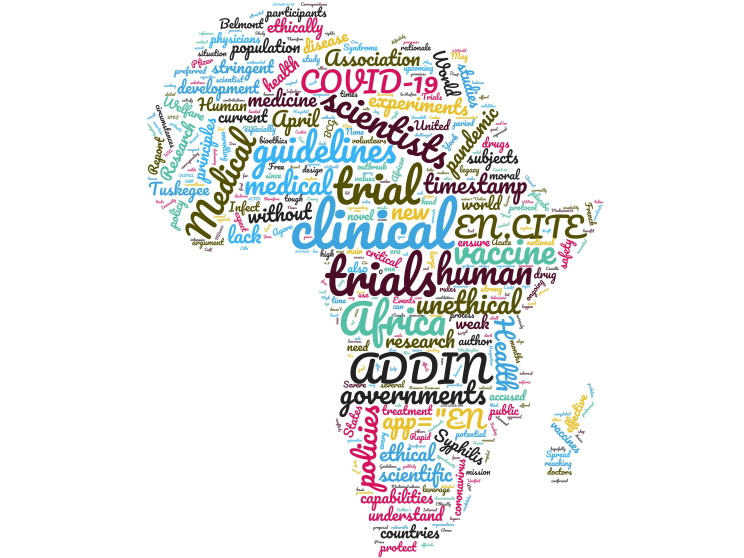
Photo: From the author's own collection, used with permission.

For instance, on 1 April 2020, on the French national television “The News Channel”, it was with worry that I heard Dr. Camille Locht, the head of the Center for Infection and Immunity of Lille and Dr. Jean Paul Mira, the head of the intensive care at the Cochin Hospital in Paris, publicly discussing about conducting vaccine clinical trials in Africa to explore potential preventive effects of the existing vaccine BCG on COVID-19 with the main argument that Africa “anyhow” doesn’t have the capabilities to face this crisis representing therefore an opportunity to conduct experimentations using real placebo and the vaccine BCG at large scale thus reaching satisfactory statistical value [8]. The argument to target Africa for clinical trials because of weak capabilities such as the lack of masks, treatments and ventilators systems is ethically unacceptable. Some could ask why to prioritize Africa in clinical trials while it was not the epicenter of the disease?

Although these are uncertain times for all of us with limited resources and capacities, public health officials, policy makers, researchers, and physicians should place human at the center of their decision-making processes. This period should not be the opportunity to discriminate some of the world population, as one may think that clinical trials could be performed on them without the same stringent measures as on other population. This is the moment for us to use our bioethics lessons and moral rationale in every decision-making process. It is ethically unacceptable to leverage the lack of capabilities and poverty to satisfy our desire to understand scientific mechanisms and examine the effectiveness of drug and vaccine candidates. Although these are tough times, scientists and physicians have to ensure that their trial study proposals are scientifically and ethically valid and respect the participant’s rights and safety. These studies should also align with the guidelines of national regulatory agencies in Africa. Additional layers of clinical trials supervision from international organizations such as the world health Organization (WHO) and the World Medical Association (WMA) could help to enforce the trial guidelines.

At the end of the day, we need clinical trials and we can understand that some scientists and medical doctors could be attracted to leverage the lack of capabilities and weak public health policies in some countries to reach satisfactory statistical values. Ethically, as researchers, we shall pursue our scientific mission against COVID-19 without enrolling exploitatively vulnerable population to clinical trials for the benefit of others.

## CONCLUSION

During this pandemic era, unethical trial might occur only if the policy and the surveillance of the trial are weak. Therefore, the African governments, the African Union, the WHO, and the WMA should coordinate their effort to ensure a transparent process of ongoing and upcoming clinical trials in Africa. It is also the time for Africa governments to draft a unique and stringent continental guideline for clinical trials and a detailed protocol to assess clinical studies validities. The policymakers and governments should take a close look at clinical trial proposals to avoid potential unethical scientific experiments. Although a critical time, as scientist, we cannot afford to repeat the dark side of clinical trials legacy.
